# Mechanisms and genetic mutations of pyrethroid resistance in *Aedes albopictus* in the context of urbanization: a case study of Hangzhou, China

**DOI:** 10.3389/fcimb.2025.1566942

**Published:** 2025-03-25

**Authors:** Binbin Jin, Lingya Wei, Tianxiao Duan, Yinghong Wang, Huimin Wang, Hui Jin

**Affiliations:** ^1^ Institute of Disinfection and Vector Control, Hangzhou Center for Disease Control and Prevention (Hangzhou Health Supervision Institution), Hangzhou, Zhejiang, China; ^2^ Zhejiang Key Laboratory of Multi-Omics in Infection and Immunity, Hangzhou, Zhejiang, China; ^3^ Department of Quality Management, Hangzhou Center for Disease Control and Prevention (Hangzhou Health Supervision institution), Hangzhou, Zhejiang, China

**Keywords:** *Ae. albopictus*, pyrethroid resistance, *VGSC*, mutation, urbanization

## Abstract

**Background:**

The Asian tiger mosquito (*Aedes albopictus*) serves as a globally significant vector for arboviruses such as dengue, chikungunya, and Zika. The extensive application of pyrethroid insecticides has led to a growing resistance in *Ae. albopictus* populations, thereby compromising mosquito control initiatives. This study examines the mechanisms underlying pyrethroid resistance and the related genetic mutations in *Ae. albopictus* within the framework of urbanization, with the objective of informing the development of effective control strategies.

**Methods:**

*Ae. albopictus* larvae were sampled from five districts in Hangzhou, China, each characterized by different levels of urbanization. Resistance to beta-cypermethrin and permethrin were evaluated utilizing the World Health Organization (WHO) tube test methodology. Molecular analyses were conducted to identify mutations in the *voltage-gated sodium channel* (*VGSC*) gene, with a specific focus on the F1534S mutation. The data were subjected to statistical analysis using Fisher’s exact test, chi-square test, and Pearson correlation to assess the relationship between resistance levels and urbanization.

**Results:**

Populations of *Ae. albopictus* in Hangzhou demonstrated substantial resistance to pyrethroids, with mortality rates falling below 90%. Notably, the Binjiang District exhibited the lowest mortality rates, with 20.55% for beta-cypermethrin and 21.21% for permethrin, whereas Chun’an County displayed relatively higher mortality rates of 32.00% and 47.28%, respectively. The F1534S mutation was predominantly observed, with homozygous (S/S) mutations constituting 87.78% and 83.29% of the populations exposed to beta-cypermethrin and permethrin, respectively. Chi-square analyses confirmed a significant association between the F1534S mutation and resistance (*P* < 0.01). Furthermore, no significant correlation was identified between resistance levels and urbanization rates (*P* > 0.05), indicating that urbanization is not a primary factor contributing to resistance.

**Conclusion:**

The F1534S mutation is pivotal in conferring pyrethroid resistance in *Ae. albopictus*. To enhance the effectiveness of mosquito control strategies, it is imperative to incorporate resistance monitoring, insecticide rotation, and non-chemical approaches. Additionally, further research is warranted to investigate alternative resistance mechanisms and the influence of urbanization on mosquito ecology.

## 1 Background

The Asian tiger mosquito (*Aedes albopictus*) is a vector species of global significance, capable of transmitting several arboviruses, including dengue, chikungunya, and Zika viruses, thereby posing a substantial threat to global public health (Bonizzoni et al., 2013). In China, the incidence of mosquito-borne diseases such as dengue fever has increased, with southern provinces like Guangdong and Yunnan reporting 83.85% of national dengue cases between 2005 and 2023 (Li et al., 2024). The widespread distribution of *Ae. albopictus* across China underscoring its pivotal role as a primary vector (Wu et al., 2010; Li et al., 2014).

In the absence of a safe and effective dengue vaccine, chemical control remains the predominant strategy for managing mosquito populations and preventing mosquito-borne diseases (Kayesh et al., 2023; Leblebicioglu et al., 2024). However, the prolonged and improper use of chemical insecticides has led to widespread insecticide resistance in mosquitoes and posed potential risks to human health and ecosystems. Pyrethroid insecticides are the most extensively utilized class due to their low human toxicity and limited ecological disruption, particularly in adult-targeted control programs (Su et al., 2019). Furthermore, the resistance levels of *Ae. albopictus* to various insecticide types vary due to regional differences in insecticide application (Li et al., 2018; Zheng et al., 2022; Liu Q, 2024). Recent nationwide surveys in China indicate that pyrethroid resistance in this species exhibits a distinct latitudinal gradient, with mortality rates dropping below 50% in southern provinces (e.g., Guangdong and Yunnan) where dengue is endemic, compared to 60–80% in northern regions (Wei et al., 2021). This disparity is strongly linked to the high-frequency occurrence of the *kdr* mutation F1534S, which is prevalent in 50–85% of southern populations versus 10–30% in northern populations (Wei et al., 2021).

The development of insecticide resistance in mosquitoes is primarily driven by genetic variation and environmental selection pressures (Liu, 2015). When mosquitoes carry mutations that confer insecticide tolerance, they exhibit higher survival rates upon exposure and transmit resistance genes to offspring, accelerating the emergence of resistant populations (Andreazza et al., 2021). Pyrethroids exert their insecticidal effects by targeting voltage-gated sodium channels (VGSC) in mosquitoes. Mutations in the *VGSC* gene can result in pyrethroid resistance, a phenomenon referred to as knockdown resistance (*kdr*). In recent years, multiple *kdr*-associated *VGSC* mutations, such as V1016G, I1532T, and F1534S/L/C, have been widely detected in *Ae. albopictus* populations across China (Zhou et al., 2019; Chen et al., 2021; Shan et al., 2023; Zhao et al., 2023). These mutations significantly diminish the efficacy of pyrethroid insecticides, thereby compromising traditional control measures. Thus, understanding the distribution of *kdr* mutations and resistance levels in *Ae. albopictus* populations across different regions is essential for advancing scientific research and optimizing public health initiatives.

Regional economic and urbanization disparities significantly influence insecticide use, thereby accelerating the development of *Ae. albopictus* resistance (Ortiz et al., 2021; Fu, 2024). Thus, examining resistance status and molecular mechanisms across regions are crucial for effective mosquito-borne disease control. Urbanization, a global trend, alters *Ae. albopictus* behavior and resistance through environmental modifications such as habitat fragmentation. For example, Hangzhou’s population of 12.52 million, with an average temperature of 15 - 23°C and 1374.9 mm annual rainfall, provides optimal conditions for *Ae. albopictus* proliferation. Consequently, urbanization level variations lead to varied mosquito resistance to identical insecticides.

This study examines five districts in Hangzhou (Gongshu, Binjiang, Qiantang, Fuyang, and Chun’an) to assess how urbanization gradients affect *Ae. albopictus* population dynamics and determine whether urban-rural disparities influence pyrethroid susceptibility. Additionally, the research analyzes associated genetic mutations, focus on the *VGSC* gene. The findings are expected to advance evidence-based strategies for mosquito control.

## 2 Materials and methods

### 2.1 Source and rearing of adult *Ae. albopictus* mosquitoes

**Figure 1:**
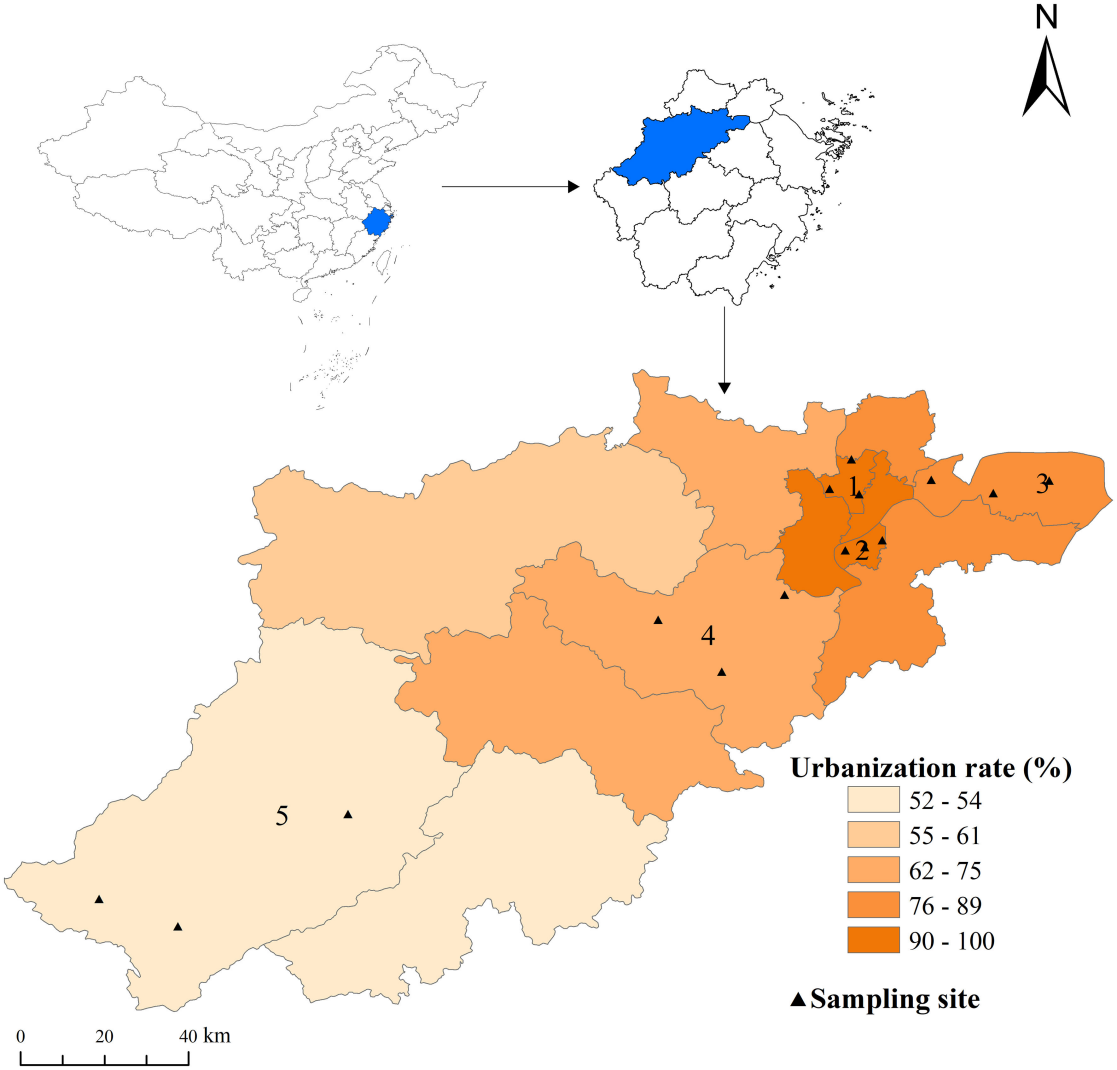
The study sites and Urbanization rates in Hangzhou, China. Study sites: (1) GongShu, urbanization rate 100%, traditional main city; (2) BinJiang, urbanization rate 100%, modern main city; (3) QianTang, urbanization rate 88.4% (4) FuYang urbanization rate 72.4%, and (5) Chun’an urbanization rate 52.3%. The black triangles represent the sampling points, with three residential areas selected in each administrative district. The map was created using ArcMap10.7.

Larvae of natural populations of *Ae. albopictus* were collected from urban residential areas across five districts in Hangzhou from May to June 2023. Selection of districts was based on the city’s urbanization gradient, ranging from fully urbanized historic cores (Gongshu and Binjiang Districts) to semi-urbanized development zones (Qiantang and Fuyang Districts) and rural areas (Chun’an County). Within each district, sampling sites were evenly distributed among three streets, with at least 50 habitats sites selected for larval collection (see **Figure 1**; **Supplementary Table 1** for details). The larvae were transported to the mosquito rearing facility at the Hangzhou Center for Disease Control and Prevention. In the facility, larvae were housed in plastic containers and fed ground turtle food (INCH-GOLD, Shenzhen, China). Environmental conditions were controlled at 27 ± 2°C, 70% ± 10% relative humidity and a 14:10-hour light-dark cycle. Adult mosquitoes were housed in 30 x 30 x 30 cm nylon mesh cages, where they were provided with a 10% glucose solution via cotton wicks and permitted to mate freely. Female mosquitoes received blood meals using defibrinated sheep blood (Solarbio Life Sciences, Beijing, China) through a Hemotek membrane feeding system (Discovery Workshops, Accrington, UK).

### 2.2 Insecticide resistance bioassays

According to the World Health Organization Pesticide Evaluation Scheme (WHOPES) (Organization W H, 2011), two categories of pyrethroid insecticides are recommended: Type I pyrethroid (permethrin, 0.25%) and Type II pyrethroid (deltamethrin, 0.03%). Following to the WHO standard tube test protocol (Organization W H, 2016a; Organization W H, 2016b), with modifications, we conducted bioassays as follows: twenty females per replicate (n=3) were transferred to WHO acrylic tubes (12 cm length × 3.5 cm diameter) lined with diagnostic-dose papers (supplied by the Chinese CDC). Control groups received silicone oil-treated papers. After 1-hour exposure, mosquitoes were gently transferred to recovery tubes using soft-bristle brushes and provided 10% glucose solution for 24 hours. Mortality was assessed at 24-hour post-recovery; individuals unresponsive to mechanical stimulation were recorded as dead. Tests were discarded if control mortality exceeded 5%, and Abbott’s was used to correct mortality rates. Post bioassay, deceased and surviving mosquitoes were separated and preserved in 95% ethanol for subsequent DNA analysis.

### 2.3 DNA extraction and *kdr* mutation detection

Genomic DNA was extracted from individual mosquitoes using a Tissue DNA Extraction Kit (magnetic bead method, Jiangsu Shuoshi Biotechnology Co., Ltd.) following the manufacturer’s protocol, with the following quality controls: each batch included blank controls and positive controls (wild type *Ae.albopictus* DNA). DNA purity (A260/A280: 1.8–2.0) was verified prior to storage at -20°C or PCR analysis. DNA extraction was performed on all mosquitoes subjected to resistance testing, encompassing both surviving and deceased specimens, to screen for insecticide resistance-linked mutations in target genes. The extracted DNA was stored at −20°C or directly used for PCR. Primers, as designed by Kasai (Kasai et al., 2011), were employed to amplify specific fragments of the *VGSC* gene domains II to IV, using 2×DreamTaq Green PCR Master Mix (Thermo Fisher) with genomic DNA from individual mosquitoes serving as the template. The amplified fragments covered domain II, which includes sites S989, I1011, L1014, and V1016; domain III, which includes sites I1532 and F1534; and domain IV, which includes site D1763, as detailed in **Table 1**. The PCR products were subsequently analyzed via 1% agarose gel electrophoresis, and samples exhibiting distinct bands without smearing were selected for Sanger sequencing. Primer synthesis and Sanger sequencing were conducted by Shanghai Sangon Biotech Co., Ltd. Sequence alignment was conducted using Mega 11.0.13 software, and sequencing chromatograms were analyzed using Chromas 2.6.6 software.

**Table 1:** Amplification products and reaction conditions for *Ae. albopictus VGSC* gene fragments.

Amplify fragments	Primer name	Mutation sites	Primer sequences 5’ - 3’	Annealing temperature (°C)	Product length (bp)
Domains II	acgSCF20	S989、I1011、L1014、V1016	GACAATGTGGATCGCTTCCC	55	480
	acgSCR21		GCAATCTGGCTTGTTAACTTG		
Domains III	acgSCF7	I1532、F1534	GAGAACTCGCCGATGAACTT	53	740
	acgSCR7		GACGACGAAATCGAACAGGT		
Domains IV	acgSCF6	D1763	TCGAGAAGTACTTCGTGTCG	55	280
	acgSCR8		AACAGCAGGATCATGCTCTG		

### 2.4 Statistical analysis

Resistance status was determined based on mortality rates: mortality less than 90% indicated resistance; mortality between 90% and 98% suggested possible resistance; and mortality greater than 98% indicated susceptibility (Organization W H, 2016c). To assess associations between nonsynonymous mutations and resistance, Fisher’s exact test or the *χ* ^2^ test (when all n > 5) was used, with tests were Bonferroni correction for multiple comparisons. Odds ratios (*ORs*) was calculated for each mutation. One-way ANOVA was used to compare insecticide resistance and mean pupation time across research locations and the control group. Pearson correlation analysis was conducted to explore the relationship between pupation time in different urban districts and the level of urbanization, as well as the relationship between mean insecticide resistance and the level of urbanization. Prior to correlation analyses, the normality of continuous variables was assessed using Shapiro-Wilk tests. Variables violating normality assumptions (*p* < 0.05) were analyzed with Spearman’s rank correlation coefficient. A significance level of *p* < 0.05 was considered statistically significant for all analyses.

## 3 Result

### 3.1 The influence of urbanization on the pupation duration of *Ae. albopictus* and its correlation with the rate of urbanization

**Figure 2:**
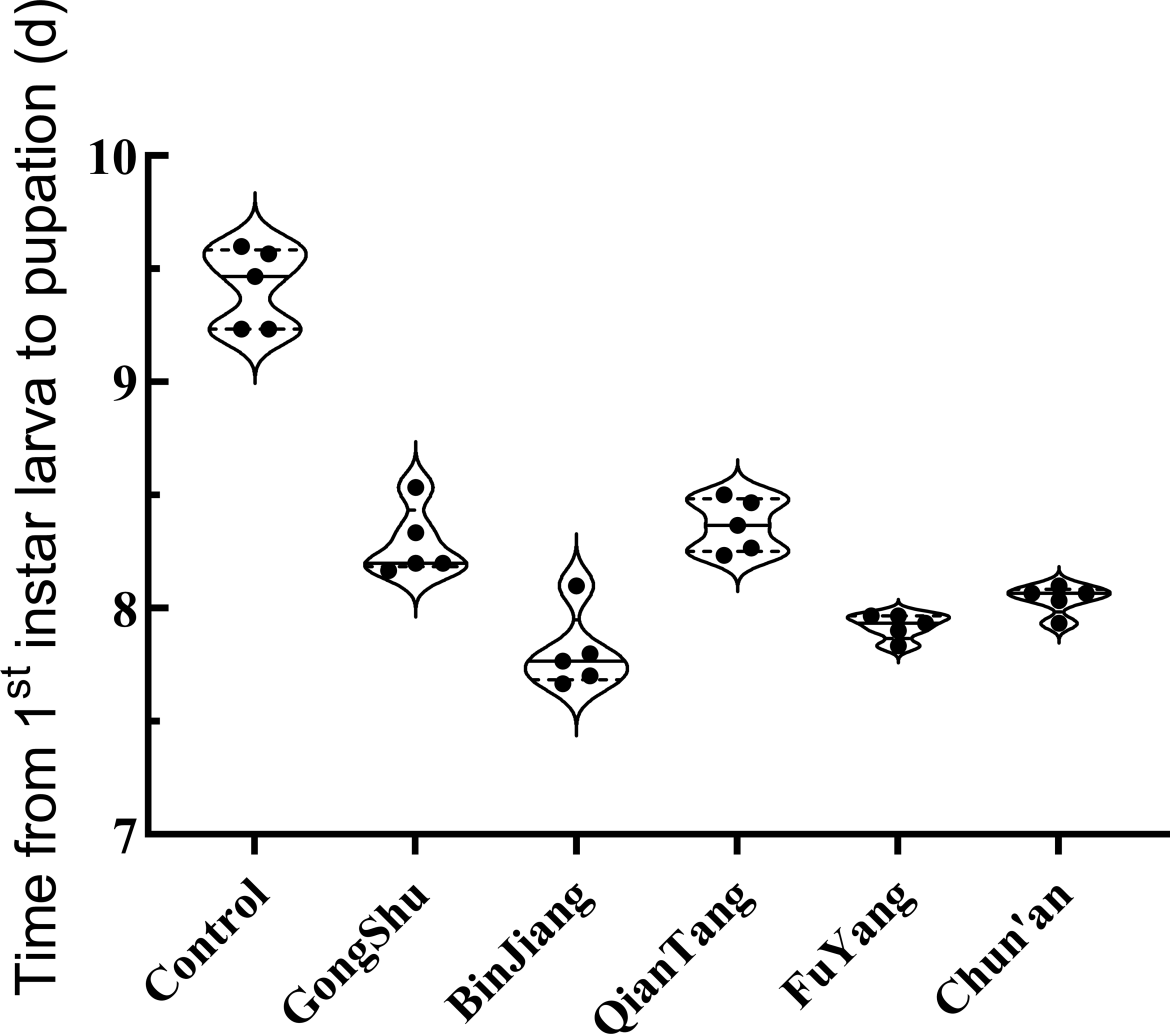
Development duration from first Instar to pupation of *Ae. albopictus* in various districts of Hangzhou. This graph delineates the time required for *Ae. albopictus* to transition from the first instar larval stage to pupation across different districts in Hangzhou. Each data point signifies the mean development duration, measured in days, for a particular district. The study involved 30 larvae per group, with a total of five replicates.

The study identified significant variations in pupation times among different districts and the control group (*F* = 87.36, *P* < 0.001). The mean pupation times were notably shorter in urban districts, ranging from 7.81 to 8.37 days, compared to the control group, which exhibited a mean pupation time of 9.42 days (refer to **Figure 2** for detailed data). A *post-hoc* Tukey’s HSD test revealed that the control group had significantly longer pupation times than all urban districts (*P* < 0.05), while no significant differences were observed among the districts themselves.

To investigate the relationship between urbanization rate and pupation time, a linear regression analysis was conducted. The analysis indicated a non-significant trend towards reduced pupation times with increasing urbanization rates (*R²* = 4.33%, *p* = 0.729). These findings suggest that the urbanization rate may exert minimal influence on the developmental rate of *Ae. albopictus* larvae. However, further research with a larger sample size may be required to substantiate this relationship.

### 3.2 Ae. albopictus has developed significant resistance to currently utilized pyrethroid insecticides

**Figure 3:**
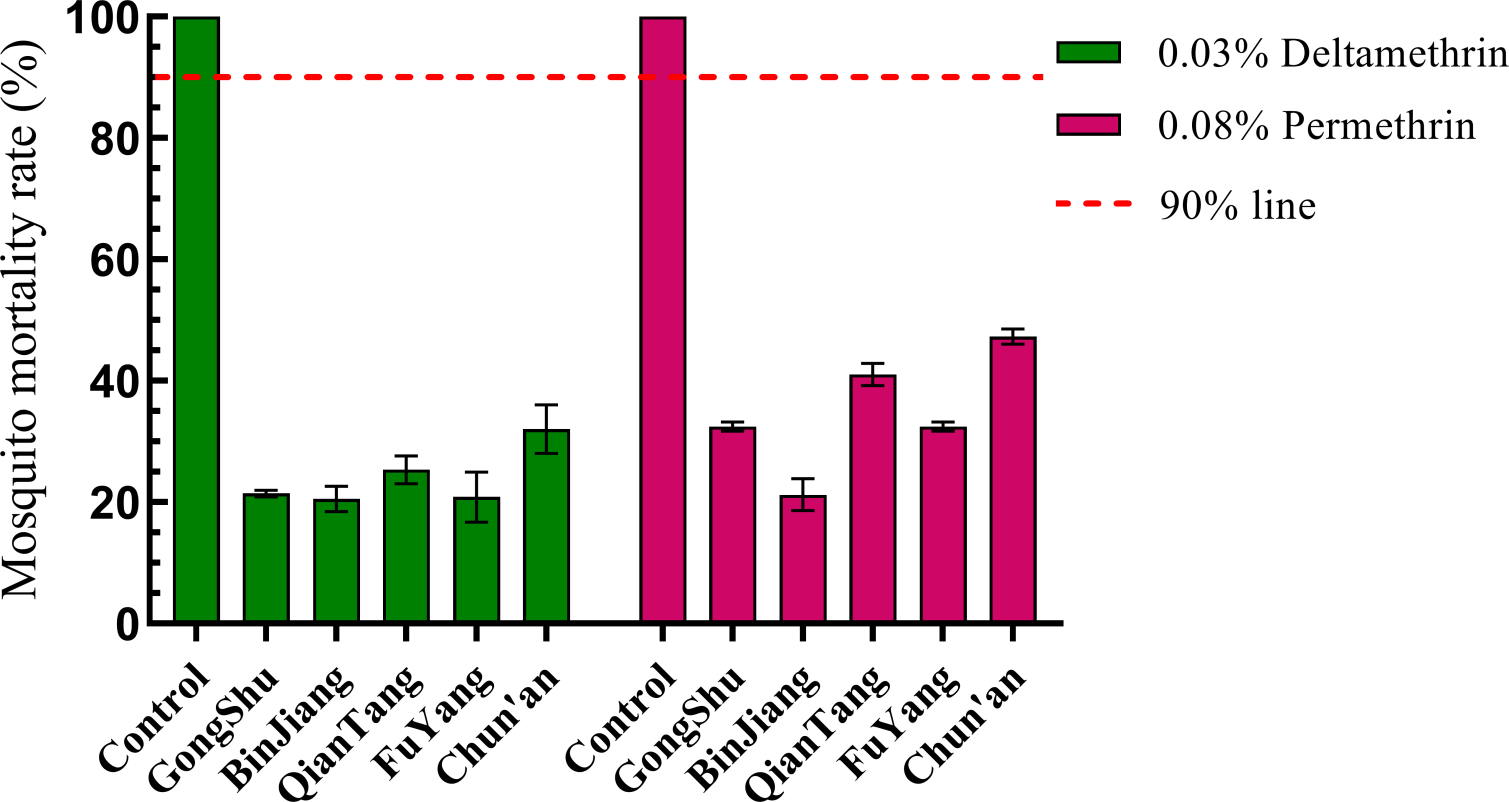
Illustrates the resistance levels of *Ae. albopictus* to contemporary insecticides across various administrative districts in Hangzhou, 2023. Populations exhibiting mortality rates below 90% are classified as resistant. The error bars represent the standard deviation (SD).

Adult populations of *Ae. albopictus* from five distinct regions demonstrated resistance to two specific pyrethroid insecticides, beta-cypermethrin and permethrin, with mortality rates falling below 90% (**Figure 2**). Notably, the Binjiang population exhibited the lowest mortality rates for beta-cypermethrin and permethrin, at 20.55% and 21.21%, respectively, whereas the Chun’an population displayed the highest mortality rates, at 32.00% and 47.28%. Mortality rates in the remaining regions were intermediate between these two extremes (**Figure 3**). The observed differences in resistance levels across the regions were not statistically significant (*F* = 1.002, *P* = 0.396), nor was there a statistically significant correlation between resistance levels and the rate of urbanization (correlation coefficient *R* = -0.776, *P* = 1.123). Although the negative correlation coefficient suggests a potential inverse relationship between resistance and urbanization level, the *P*-value exceeding 0.05 indicates that this association is not statistically significant.

### 3.3 The mutation at codon 1534 of the *VGSC* gene is correlated with resistance to pyrethroid insecticides

Across all administrative districts, no mutations were detected at positions 989, 1011, 1014, and 1016 in domain II, position 1532 in domain III, or position 1763 in domain IV of the *VGSC* gene in *Ae. albopictus* samples exposed to insecticides. However, a substantial number of mutations were identified at position 1534 in domain III (see **Tables 2**, **3**). At this position, two alleles were discerned: the wild-type TTC (F), which encodes phenylalanine, and the mutant-type TCC (S), which encodes serine. Three genotypes were observed: wild-type homozygous F/F, wild-type/mutant heterozygous F/S, and mutant homozygous S/S.

**Table 2:** *Kdr* mutations at position F1534 of the *VGSC* gene in *Ae. albopictus* exposed to deltamethrin.

Location	Phenotype	N	Genotype			Allele frequency (%)		*P*	Odds Ratio (95%CI)
			F/F	F/S	S/S	TTC(F)	TCC(S)		
GongShu	S	15	3 (20.00)	1 (6.67)	11 (73.33)	23.33%	76.67%	<0.001	9.6580 (2.7955, 33.3670)
	R	55	0 (0)	3 (5.45)	52 (94.55)	2.73%	97.27%
BinJiang	S	14	2 (14.29)	1 (7.14)	11 (78.57)	17.86%	82.14%	0.008	4.1707 (1.4831, 11.7286)
	R	54	0 (0)	5 (9.26)	49 (90.74)	4.63%	95.37%
QianTang	S	19	3 (15.79)	2 (10.53)	14 (73.68)	21.05%	78.95%	<0.001	6.3797 (2.1026, 19.3574)
	R	56	0 (0)	5 (8.93)	51 (91.07)	4.46%	95.54%
FuYang	S	15	3 (20.00)	2 (13.33)	10 (66.67)	26.67%	73.33%	<0.001	8.8767 (2.9746, 26.4893)
	R	57	0 (0)	4 (7.02)	53 (92.98)	3.51%	96.49%
Chun'an	S	24	4 (16.67)	2 (8.33)	18 (75.00)	20.83%	79.17%	0.004	4.1646 (1.6020, 10.8261)
	R	51	2 (3.92)	2 (3.92)	47 (92.16)	5.88%	94.12%

**Table 3:** *Kdr* mutations at position F1534 of the *VGSC* gene in *Ae. albopictus* exposed to permethrin.

Location	Phenotype	N	Genotype			Allele frequency (%)		P	Odds Ratio (95%CI)
			F/F	F/S	S/S	TTC(F)	TCC(S)		
GongShu	S	24	3 (12.50)	4 (16.67)	17 (70.83)	20.83%	79.17%	<0.001	6.3797 (2.1026, 19.3574)
	R	50	1 (2.00)	2 (4.00)	47 (94.00)	4.00%	96.00%
BinJiang	S	14	2 (14.29)	1 (7.14)	11 (78.57)	17.86%	82.14%	<0.001	10.7561 (2.4240, 47.7286)
	R	52	0 (0)	2 (3.85)	50 (96.15)	1.92%	98.08%
QianTang	S	23	3 (13.04)	3 (13.04)	17 (73.91)	19.57%	80.43%	0.004	4.6000 (1.6508, 12.8178)
	R	33	0 (0)	3 (9.09)	29 (87.88)	4.55%	92.42%
FuYang	S	25	5 (20.00)	3 (12.00)	17 (68.00)	26.00%	74.00%	0.003	3.5526 (1.5682, 8.0480)
	R	52	1 (1.92)	7 (13.46)	44 (84.62)	8.65%	91.35%
Chun'an	S	35	7 (20.00)	5 (14.29)	23 (65.71)	27.14%	72.86%	0.001	4.2534 (1.8240, 9.9185)
	R	39	1 (2.56)	4 (10.26)	34 (87.18)	7.69%	92.31%

In adult *Ae. albopictus* specimens subjected to deltamethrin, heterozygous mutations (F/S genotype) at position 1534 of the *VGSC* gene constituted 7.50% (27/360), whereas homozygous mutations (S/S genotype) comprised 87.78% (316/360) (refer to **Table 2**). Similarly, among those exposed to permethrin, heterozygous mutations (F/S genotype) at the same position accounted for 9.80% (34/347), and homozygous mutations (S/S genotype) represented 83.29% (289/347) (refer to **Table 3**). Chi-square analyses indicated statistically significant differences in the prevalence of resistance-associated mutations between susceptible and resistant phenotypes to pyrethroid insecticides (all *P* < 0.01). The Odds Ratio (OR) exceeded 1 across all sampling locations, signifying that the TCC (S) allele was significantly more prevalent in resistant individuals compared to the TTC (F) allele in all regions. This finding suggests an association between the TCC (S) mutation and resistance to pyrethroid insecticides.

## 4 Discussion

The findings of this study offer significant insights into the mechanisms underlying pyrethroid resistance in *Ae. albopictus* and the potential impact of urbanization on the evolution of such resistance. The results demonstrate that *Ae. albopictus* populations in Hangzhou, China, have developed substantial resistance to commonly employed pyrethroid insecticides, including beta-cypermethrin and permethrin. This resistance is closely linked to specific mutations in the *VGSC* gene, notably the F1534S mutation, which has been consistently identified in resistant populations across all examined districts.

### 4.1 Resistance levels and urbanization

The investigation demonstrated that *Ae. albopictus* populations across all five districts in Hangzhou exhibited resistance to pyrethroid insecticides, as evidenced by mortality rates consistently falling below 90%. Although variations in resistance levels were observed among the districts, these differences did not reach statistical significance, and no definitive correlation was identified between urbanization rates and resistance levels. This indicates that urbanization, despite its potential impact on mosquito habitats and breeding conditions, may not be the primary factor driving pyrethroid resistance in *Ae. albopictus*. In addition, insecticide use homogeneity, i.e., the main components of the insecticides used in different regions are similar, limiting variations in selection pressures, and the ubiquitous urban heat island effect can mitigate the temperature-related cost of resistance, allowing resistance genotypes to persist even in sub-optimal habitats. Rather, the pervasive application of pyrethroid insecticides in both urban and semi-urban areas likely imposes a uniform selective pressure, fostering the development of resistance across diverse regions.

### 4.2 Genetic mutations and resistance mechanisms

The prevalence of the F1534S mutation in the *VGSC* gene among resistant *Ae. albopictus* populations highlights the critical role of this genetic alteration in conferring resistance to pyrethroids. The F1534S mutation, characterized by the substitution of phenylalanine with serine, has been previously associated with decreased sensitivity to pyrethroids across various mosquito species, including *Ae. albopictus* and *Ae. aegypti* (Kasai et al., 2011; Xu et al., 2016; Kawada et al., 2020; Kushwah et al., 2020; Zhao et al., 2023). In the present study, the F1534S mutation was identified in both heterozygous (F/S) and homozygous (S/S) forms, with the latter being significantly more prevalent among resistant individuals. This observation corroborates previous research that has established the S/S genotype as a robust indicator of pyrethroid resistance (Chen et al., 2021; Zheng et al., 2022).

The absence of mutations at other frequently reported *kdr* sites, such as V1016G and I1532T, suggests that the F1534S mutation may constitute the primary genetic mechanism underpinning pyrethroid resistance in *Ae. albopictus* populations in Hangzhou. Nonetheless, further investigation is warranted to examine the potential involvement of alternative resistance mechanisms, such as metabolic detoxification, which may also contribute to the observed resistance patterns. In future practice, it may be considered to use chemical pesticides together with corresponding synergists to enhance the effect of pesticides.

### 4.3 Implications for mosquito control

The elevated levels of pyrethroid resistance identified in this study have profound implications for mosquito control strategies in Hangzhou and other regions where *Ae. albopictus* serves as a primary vector of arboviruses. The extensive application of pyrethroids, particularly in urban settings, has likely accelerated the development of resistance, thereby diminishing the efficacy of these insecticides in controlling mosquito populations. This situation underscores the urgent necessity for alternative control measures, such as the rotation of insecticide classes, the implementation of non-chemical methods (e.g., biological control or environmental management), and the development of new insecticides with novel modes of action (Zhao et al., 2023).

Furthermore, the identification of the F1534S mutation as a critical resistance marker offers a valuable tool for monitoring resistance levels in *Ae. albopictus* populations. Regular surveillance of *kdr* mutations can inform decision-making concerning insecticide application and resistance management strategies, ultimately enhancing the effectiveness of mosquito control programs.

### 4.4 Limitations and future directions

This study provides critical insights into pyrethroid resistance in *Ae. albopictus*; however, several limitations require consideration in future research. First, the exclusive focus on Hangzhou may limit generalizability to regions with divergent ecological profiles (Shan et al., 2023; Zhao et al., 2023). Expanding geographic sampling and assessing environmental variable impacts should be prioritized. Second, while kdr mutations were thoroughly examined, other mechanisms like metabolic resistance (e.g., CYP450-mediated detoxification via RNA sequencing/proteomics) or behavioral adaptations (Liu, 2015) remain underexplored. Future work must quantify the relative contributions of these pathways. Third, urbanization’s behavioral effects—including feeding rhythm alterations and habitat selection—were not empirically tested despite their potential resistance implications (Andreazza et al., 2021).

In conclusion, this work demonstrates that Hangzhou’s Ae. albopictus populations exhibit substantial pyrethroid resistance, primarily linked to the *VGSC* gene F1534S mutation. Although urbanization modulates mosquito breeding ecology, it appears secondary to genetic factors in resistance development. These results underscore the urgency of implementing integrated vector management combining resistance surveillance, insecticide rotation protocols, and novel control technologies to mitigate arbovirus transmission risks.

## Data Availability

The datasets presented in this study can be found in online repositories. The names of the repository/repositories and accession number(s) can be found in the article/**Supplementary Material**.
